# Honey as a Potential Natural Anticancer Agent: A Review of Its Mechanisms

**DOI:** 10.1155/2013/829070

**Published:** 2013-12-02

**Authors:** Sarfraz Ahmed, Nor Hayati Othman

**Affiliations:** Department of Pathology, School of Medical Sciences, Universiti Sains Malaysia, 16150 Kubang Kerian, Kelantan, Malaysia

## Abstract

The main treatment for cancer is by using chemotherapy and radiotherapy which themselves are toxic to other viable cells of the body. Recently, there are many studies focusing on the use of natural products for cancer prevention and treatment. Of these natural products, honey has been extensively researched. The mechanism of the anti-cancer activity of honey as chemopreventive and therapeutic agent has not been completely understood. The possible mechanisms are due to its apoptotic, antiproliferative, antitumor necrosis factor (anti-TNF), antioxidant, anti-inflammatory, estrogenic and immunomodulatory activities. We collate the findings of several studies published in the literature in order to understand the mechanism of its action.

## 1. Introduction

Annually cancer is diagnosed in approximately 11 million people causing 7.6 million deaths worldwide [1]. Cancer is a multistep process. It starts as an onset from a single transformed cell. Its genesis is characterized by the swift proliferation, invasion, and metastasis [2]. This dynamic process is activated by various carcinogens, tumor promoters, and inflammatory agents. The whole modulation is controlled through the transcription factors, proapoptotic proteins, antiapoptotic proteins, protein kinases, cell cycle proteins, cell-adhesion molecules, cyclooxygenase-2 (COX-2), and other molecular targets [2–4].

The standard treatments against cancer are surgery, radiotherapy, and chemotherapy. These modalities are beset with serious side effects [5]. New targets for cancer treatment focus on interfering with specific targeted molecules needed in carcinogenesis [6].

Natural products such as honey have potential anticancer effect [7]. Honey is composed of various sugars, flavonoids, phenolic acids, enzymes, amino acids, proteins, and miscellaneous compounds (Table 1). Its composition varies according to floral sources and origin [8]. It has been shown to have anti-inflammatory [9], antimicrobial [10], antimutagenic [11], antioxidant [12], and antitumor [7, 13, 14] effects. The phenolic contents of honey have been reported to have antileukemic activity against different types of leukemic cell lines [15]. Its anticancer activity has been proved against various cancer cell lines and tissues, such as breasts [14, 16–19], colorectal [20], renal [21], prostate [17], endometrial [17], cervical [19] and oral cancer [22].

Honey potentiates the antitumor activity of chemotherapeutic drugs such as 5-fluorouracil and cyclophosphamide [18]. Studies exhibiting anticancer effect of honey range from tissue cultures [17, 19, 20, 22, 23] and animal models [14, 16, 18, 24] to clinical trials [25]. Polyphenols in honey are considered as one of the main factors responsible for the anticancer activity of honey [15, 26].

This review presents current progress in understanding the mechanism of anticancer activity of honey.

## 2. Honey and Its Apoptotic Activity

Two characteristics of cancer cells are uncontrolled cellular proliferation and inadequate apoptotic turnover [27]. Drugs which are commonly used for cancer treatment are apoptosis inducers [28]. Programmed cell death or apoptosis is categorized into three phases: (a) an induction phase, (b) an effector phase, and (c) a degradation phase [29]. The induction phase stimulates proapoptotic signal transduction cascades through death-inducing signals. Effector phase is committed to bring cell death via a key regulator, mitochondrion. The last degradation phase comprises nuclear and cytoplasmic events. Nuclear change includes chromatin and nuclear condensation, cell shrinkage, DNA fragmentation, and membrane blebbing [28, 29]. In the cytoplasm, a complex cascade of protein cleaving enzymes called caspases is activated. The cell is finally destined into fragmented apoptotic bodies which are phagocytosed by macrophages or other surrounding cells [28, 29].

**Figure 1:**
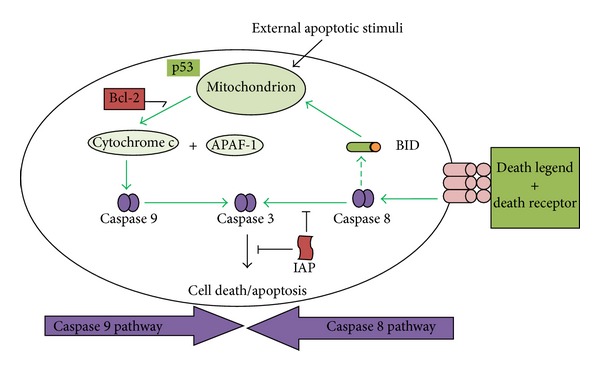
Apoptosis following caspase 8 and caspase 9 pathways; source [30]. *Bcl-2: B cell lymphoma 2 protein; Bid: Bcl-2 associated X proteins; Cyt. C: cytochrome C; Apaf-1: apoptotic protease activating factor; IAP: inhibitor of apoptosis proteins; Caspase 3-caspase protein that interacts with caspase 8 and caspase 9. *

The apoptosis usually follows two pathways: the caspase 8 or death-receptor pathway and caspase 9 or mitochondrial pathway [30] (Figure 1).

**Figure 2:**
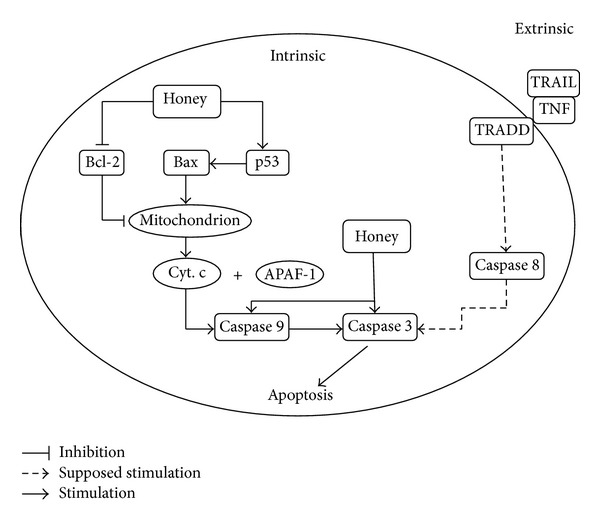
The effect of honey on apoptotic pathway. Honey exerts apoptotic effect through upregulation and modulation of proapoptotic proteins (p53, Bax, caspase 3, and caspase 9) and downregulation of antiapoptotic proteins (Bcl-2) [14, 23, 31]. *Bcl-2: B cell lymphoma 2 protein; Cyt. C: cytochrome C; Apaf-1—apoptotic protease activating factor 1; TNF: tumor necrosis factor; TRAIL: TNF related apoptosis-inducing ligand; TRADD: TNFR associated death domain protein. *

Honey induces apoptosis in various types of cancer cells via depolarization of mitochondrial membrane [19]. Honey elevates caspase 3 activation level and *poly (ADP-ribose) polymerase (PARP) *cleavage in human colon cancer cell lines [20] which is attributed to its high tryptophan and phenolic content [20]. It also induces apoptosis by upregulating and modulating the expression of pro- and antiapoptotic proteins in colon cancer cell lines [23]. Honey increases the expression of caspase 3, p53, and proapoptotic protein Bax and downregulates the expression of antiapoptotic protein Bcl2 [23] (Figure 2). Honey generates ROS (reactive oxygen species) resulting in the activation of p53 and p53 in turn modulates the expression of pro- and antiapoptotic proteins like Bax and Bcl-2 [23]. Honey as an adjuvant therapy with *Aloe vera *boosts the expression of proapoptotic protein Bax and decreases the antiapoptotic protein Bcl-2 expression in Wistar rats [14]. Manuka honey exerts its apoptotic effect on cancer cells through the induction of the caspase 9 which in turn activates the caspase-3, the executor protein. Apoptosis induced by Manuka also involves induction of DNA fragmentation, activation of PARP, and loss of Bcl-2 expression [31]. The apoptotic property of honey makes it a possible natural substance as antic-cancer agent as many chemotherapeutics currently used are apoptosis inducers.

## 3. Honey and Its Antiproliferative Activity

Epithelial cell divides throughout life. The cell cycle comprises three distinguished phases known as G_0_, G_1_, S, and G_2 _M. All the events in the cell cycle are regulated and monitored by several different proteins. The control panel of cell cycle comprises cyclins and cyclin-dependent kinases. The G_1_/S phase transition is a vital regulatory point where cell's fate is destined for quiescence, proliferation, differentiation and apoptosis [32]. Overexpression and dysregulation of cell cycle growth factors such as cyclin D1 and cyclin-dependent kinases (CDK) are linked with tumorigenesis [32]. The loss of this regulation is the hallmark of cancer [32]. The nuclear protein Ki-67 is a novel marker to probe the “growth fraction” of cell proliferation. It is absent in the resting phase (G_0_) but expressed during the cell cycle in all the proliferation phases (G_1_, S, G_2_, and mitosis) [33].

Honey has been shown to affect cell cycle arrest. Administration of honey mixed with *Aloe vera* solution showed a marked decrease in expression of Ki67-LI in tumor cells in rats [14]. It suggests that honey therapy could lead to lowering tumor cell proliferation by arresting cell cycle [14].Honey and its several components (like flavonoids and phenolics) are reported to block the cell cycle of colon [20], glioma [34], and melanoma [35] cancer cell lines in G_0_/G_1_ phase. This inhibitory effect on tumor cell proliferation follows the downregulation of many cellular pathways via tyrosine cyclooxygenase, ornithine decarboxylase, and kinase [20, 34–36]. The results of 3-(4,5-dimethylthiazol-2-yl)-2,5-diphenyl tetrazolium bromide (MTT) and the trypan blue exclusion assays have confirmed that anti-proliferative effect of honey is a dose- and time-dependent manner [35]. Honey or its components mediate inhibition of cell growth due to its perturbation of cell cycle [35, 36]. Cell cycle is also regulated by p53 which is involved in tumor suppression. Honey is reported to be involved in modulation of p53 regulation [20].

## 4. Honey and Its Effect on Tumor Necrosis Factor (TNF)

Tumor necrosis factor (TNF), have been shown to mediate tumor initiation, promotion, and progression [37]. The proinflammatory effect of TNF is linked to many diseases due to its ability to activate NF-kB [38]. It activates NF-kB, leading to the expression of inflammatory genes like lipoxygenase-2 (LOX-2), cyclooxygenase-2 (COX-2), cell-adhesion molecules, chemokines, inducible nitric oxide synthase (iNOS), and inflammatory cytokines [38]. It is considered as a growth factor for many of the tumor cells [38]. In contradiction, TNF-*α* has also been shown to be involved in host defense mechanisms as a key cytokine [39]. It has been shown to play a dual role, beneficial and deleterious for the promotion or inhibition of infectious diseases [39, 40].

Royal jelly (RJ) proteins (apalbumin-1 and apalbumin-2) in honey have antitumor properties. These proteins stimulate macrophages to release cytokines TNF-*α*, interleukin-1(IL-1) and interlueken-6 (IL-6) [41, 42]. Pasture, jelly bush, and Manuka honeys (at concentrations of 1% w/v) stimulate monocytes to release tumor necrosis factor-alpha and interleukin- (IL-) 1*β* and IL-6 [43, 44]. The possible mechanism involves the binding of TNF-R to TNF-*α* and adaptor protein such as TNFR associated death domain protein (TRADD), TNF receptor associated factor (TRAF), and receptor-interacting protein (RIP) to regulate apoptosis and inflammation through these cytokines [45]. This TNF-*α* release can play a pivotal role as a key cytokine to regulate important cellular processes such as apoptosis, cell proliferation, and inflammation [41, 45].

## 5. Honey and Its Anti-Inflammatory and Immunomodulatory Activities

Chronic inflammation is linked to cancer formation. Excessive or prolonged inflammation can prevent healing by damaging tissues. Honey exhibits anti-inflammatory response [46]. The literature shows that it reduces inflammation when applied in cell cultures [47], animal models [48, 49], and clinical trials [46, 50]. The inflammatory process is induced by various types of chemicals and/or biological agents including proinflammatory enzymes and cytokines [51]. The enzyme cyclooxygenase-2 (COX-2) in inflammatory process catalyses the metabolism of arachidonic acid to prostaglandin [52, 53]. Anomalous arachidonic acid metabolism is involved in carcinogenesis and inflammation [54]. COX-2 is overexpressed in premalignant and malignant conditions [54]. Phenolic compounds in honey are responsible for anti-inflammatory activity [55]. The mechanism involves the suppression of the proinflammatory activities of COX-2 and/or inducible nitric oxide synthase (iNOS) through these phenolic compounds or flavonoids [53]. Honey and its components have been documented to be involved in regulation of proteins such as ornithine decarboxylase, tyrosine kinase, iNOS, and COX-2 [56, 57].

Manuka, Pasture, Nigerian Jungle, and royal jelly honeys are found to increase IL-1*β*, IL-6, and TNF-*α* production [16, 44, 58]. This immunomodulatory and immunoprotective activity of honey is often linked to anticancer action [16, 59]. Honey stimulates antibodies, B and T lymphocytes, neutrophils, monocytes, eosinophils, and natural killer cells (NK-cells) production during primary and secondary immune responses in tissue culture [59–62]. It has been shown that honey stimulates macrophages, T-cells, and B-cells to provoke antitumor effect [59].

Sugars when ingested are slowly absorbed resulting in the formation of short chain fatty acid (SCFA) fermentation products [63]. It is a probable mechanism that the ingestion of honey may result in SCFA formation [64]. Research has established that, either directly or indirectly, SCFA have immunomodulatory actions [65]. Thus, honey may stimulate the immune system via these fermentable sugars [66]. A sugar, nigerooligosaccharides (NOS), present in honey has been found to have immunopotentiating activity [67]. Nonsugar components of honey may also be responsible for immunomodulation [66].

## 6. Honey and Its Antioxidant Activity

The role of oxidative stress involving free radicals in the carcinogenic process is well established [68]. Reactive oxygen species (ROS) and reactive nitrogen species (RNS), such as hydroxyl radical (^•^OH) superoxide (O_2_^•−^), hydrogen peroxide (H_2_O_2_), nitric oxide (NO^•^), peroxynitrite (ONOO^−^), and others, are oxidative stress agents which damage lipids, proteins, and DNA in cells [69]. Cells exhibit defense system against oxidative damage. This defense system consists of antioxidants or oxidative protective agents such as catalase, superoxide dismutase, peroxidase, ascorbic acid, tocopherol, and polyphenols [70]. Antioxidants acting as free radical scavengers may inhibit the cancer process in vivo [70]. The exact antioxidant mechanism is unknown, but the proposed mechanism is through hydrogen donation, free radical sequestration, metallic ion chelation, flavonoids substrates for hydroxyl and superoxide radical actions [71]. The antioxidant capacity of honey contributes to the prevention of several acute and chronic disorders such as diabetes [72], inflammatory disorders [73], cardiovascular diseases [74], and cancer [75, 76]. The phenolic acids and flavonoids are responsible for the well-established antioxidant activity of honey [77].

The antitumor effect of honey may be attributed to its antioxidant activity [75, 76]. An enhanced antioxidant status with apoptosis has been observed in *hepatocellular carcinoma cells *[75]. Daily consumption of 1.2 g/kg body weight of honey has been shown to elevate the amount and the activity of antioxidant agents such as beta-carotene, vitamin C, glutathione reductase, and uric acid [60].

## 7. Honey and Its Antimutagenic Activity

Mutagenicity, the ability to induce genetic mutation, is interlinked with carcinogenicity [78]. Honey is shown to have a strong antimutagenic agent and hence has anticarcinogenic property [79]. The effect of honey on radiation (UV or *γ*) exposed *Escherichia coli* cells shows SOS response (SOS is an error prone repair pathway contributing to mutagenicity) [79]. A study was performed to knock out some important genes such as *umuC*, *recA,* and *umuD* involved in SOS mediated mutagenesis. These changes are significantly inhibited in the presence of honey confirming its strong antimutagenic effect [79]. Honeys from different floral origins exhibit inhibition of Trp-p-1 mutagenicity [11].

## 8. Honey and Its Estrogenic Modulatory Activity

Estrogen is involved in number of cancers [80]. Honey modulates estrogen by its antagonistic action. It may be useful in estrogen-dependent cancers such as breasts and endometrial cancers [17]. Estrogen receptors tie to estrogens to dimerize and then translocate into the nuclei. These complexes then bind to the specific DNA base sequences called estrogen-response elements (EREs) resulting in transcription and translation of the estrogenic effect in the targeted tissue [80]. This signaling cascade induced by estrogens may be modulated at any stage [80]. Honeys from various floral sources are reported to mediate estrogenic effects via the modulation of estrogen receptor activity [17, 81]. This effect is attributed to its phenolic content [17]. Greek honey extracts exert estrogen agonistic effect at high concentrations (20–100 lg/mL) and antagonistic effect at low concentrations (0.2–5 *μ*g/mL) [17].

## 9. Conclusion

**Figure 3:**
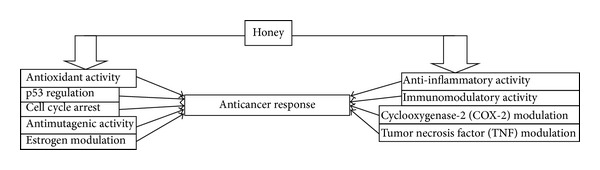
Schematic presentation of anticancer activity of honey.

Evidence is growing that honey may have the potential to be anticancer agent through several mechanisms (Figure 3). Though the full mechanism is yet to be fully understood, studies have shown that honey has anticancer effect through its interference with multiple cell-signaling pathways, such as inducing apoptosis, antiproliferative, anti-inflammatory, and antimutagenic pathways. Honey modulates the body immune system. There are still many unanswered questions; why sugar is carcinogenic, while honey which is basically sugar has anticarcinogenic properties. Honey of different floral sources may give different effects. More research is needed to improve our understanding of the positive effect of honey and cancer. What is seen in cell cultures or animal experimentations may not apply to humans. Prospective randomized controlled clinical trials are needed to validate the authenticity of honey either alone or as adjuvant therapy.

## Figures and Tables

**Table 1:** Average composition of honey-source reference, [82, 83].

Component	Value/100 g
Total carbohydrates	82.4 g
Fructose	38.5 g
Glucose	31.28 g
Sucrose	1.31 g
Maltose	7.31 g
Total acid as gluconic	0.57 g
Moisture content	17.1 g
Ash	0.169 g
Amino acids	0.3 g
Nitrogen	0.041 g
Iron	0.42 mg
Potassium	52 mg
Calcium	6.00 mg
Phosphorous	4.00 mg
Magnesium	2.00 mg
Calcium	6.00 mg
pH	3.9
